# Retraction

**DOI:** 10.1049/nbt2.12087

**Published:** 2022-05-08

## Abstract

Shahin Aghamiri, Ali Jafarpour, Mohsen Shoja: ‘Effects of silver nanoparticles coated with anti‐HER2 on irradiation efficiency of SKBR3 breast cancer cells’, IET Nanobiotechnology, 2019, 13, (8), pp. 808‐815. (https://ietresearch.onlinelibrary.wiley.com/doi/10.1049/iet‐nbt.2018.5258).

The above article, published online on 19 August 2019 in Wiley Online Library (ietresearch.onlinelibrary.wiley.com), has been retracted by agreement between the journal Editor in Chief Ronald Pethig, the Institution of Engineering and Technology, and John Wiley and Sons Ltd. The retraction has been agreed because none of the listed authors ‐ Mohsen Shoja, Ali Jafarpour and Shahin Aghamiri ‐ fulfil the journal's criteria for authorship for the research published in the article. The article was submitted for publication by Mohsen Shoja without the consent of the legitimate authors or attribution to the legitimate authors.

Shahin Aghamiri, Ali Jafarpour, Mohsen Shoja: ‘Effects of silver nanoparticles coated with anti‐HER2 on irradiation efficiency of SKBR3 breast cancer cells’, IET Nanobiotechnology, 2019, 13, (8), pp. 808‐815. (https://ietresearch.onlinelibrary.wiley.com/doi/10.1049/iet‐nbt.2018.5258).

The above article, published online on 19 August 2019 in Wiley Online Library (ietresearch.onlinelibrary.wiley.com), has been retracted by agreement between the journal Editor in Chief Ronald Pethig, the Institution of Engineering and Technology, and John Wiley and Sons Ltd. The retraction has been agreed because none of the listed authors ‐ Mohsen Shoja, Ali Jafarpour and Shahin Aghamiri ‐ fulfil the journal's criteria for authorship for the research published in the article. The article was submitted for publication by Mohsen Shoja without the consent of the legitimate authors or attribution to the legitimate authors.

